# A Framework for Long-Term Vibration-Based Monitoring of Bridges

**DOI:** 10.3390/s21144739

**Published:** 2021-07-11

**Authors:** Emrah Erduran, Frida Kristin Ulla, Lone Næss

**Affiliations:** Department of Civil Engineering and Energy Technology, Oslo Metropolitan University, Pilestredet 35, 0166 Oslo, Norway; s339529@oslomet.no (F.K.U.); s315148@oslomet.no (L.N.)

**Keywords:** long-term SHM, vibration-based, damage detection, localization, damage indicators

## Abstract

A new framework for long-term monitoring of bridges is proposed in order to negate (i) the impact of measurement uncertainties on damage detection in vibration-based structural health monitoring and (ii) the low sensitivity of damage indicators to low levels of damage. The framework is developed using three vibration-based damage indicators that have an intuitive physical correlation with damage: modal curvature, modal strain energy and modal flexibility. The article first quantifies the efficacy of these damage indicators when based on two observations, one from the undamaged state and one from the monitored state, in detecting and locating damage for different damage levels that are simulated on an 84-m long railway bridge. A long-term monitoring framework based on a new parameter defined as the frequency of the damage indicator exceeding the threshold value within a population of observations is developed. Impact of several factors including the damage location, damage indicator used in the framework, and the noise level on the success of the developed framework was investigated through numerical analysis. The new parameter, when used together with modal strain energy, was shown to provide a very clear picture of damage initiation and development over time starting from very low damage levels. Furthermore, the location of the simulated damage can be identified successfully at all damage levels and even for very high noise levels using the proposed framework.

## 1. Introduction

The bridge infrastructure throughout the world is aging rapidly. In Europe alone, a significant portion of the over one million highway bridges are on the wrong side of their design life while, as of 2017, 35% of the railway bridges are over 100 years old [1]. In addition, both highway and railway bridges are subject to traffic loads that surpass the original design loads that they were designed for leading to an acceleration in the deterioration of their structural performance. As of 2004, over 80,000 bridges in the United States, i.e., 13.7% of the total inventory of the bridges, were classified as structurally deficient [2].

Maintenance and inspection of the bridges today are mainly based on manual inspections and requires significant resources in terms of experienced and skilled engineers. As such, the detailed inspections of the bridges are generally conducted at long intervals leaving the infrastructure potentially vulnerable. Furthermore, due to their subjective nature, the outcome of a manual inspection depends on the experience and skills of the inspecting engineer rendering an objective comparison of the condition of different bridges virtually impossible. Structural Health Monitoring (SHM) techniques present an alternative to manual inspections by generating real-time and objective data about the condition of the bridges. Of the different SHM techniques, vibration-based methods provide a very attractive alternative as they are based on harvesting the readily available vibration data and provides an opportunity for damage detection at global level without prior knowledge about potential damage location and, thus, applied widely [1,3,4,5,6,7,8,9,10,11,12,13,14,15,16,17]. Other researchers worked on long-term vibration analysis of bridges and their relation to structural damage [18,19,20,21].

Vibration-based detection methods are based on detecting the changes in one or more dynamic features of the structural system between two discrete points in time; the first of these two points belonging to the known undamaged state of the structure while the damage situation at the second point in time is unknown and sought after. The dynamic features that are used for damage detection are numerous and provide different levels of damage detection based on their complexity and their sensitivity to damage. The most widely used damage detection parameters are based on either the basic modal properties of the structure (e.g., modal frequencies or mode shapes) or features derived from these basic modal parameters. The latter group was developed in order to find features that are more sensitive to damage than the basic modal parameters while retaining the clear physical principles that make these parameters attractive [22]. Among the features derived from basic modal parameters, modal curvature [23], modal strain energy [24], and modal flexibility damage indicators are amongst the most popular used within the framework of vibration-based SHM due to their physical correlation with damage, intuitiveness and simplicity [22].

These damage indicators are based on comparing two sets of modal parameters obtained from undamaged and potentially damaged states. Several articles [1,25,26], which evaluate their performance in detecting, locating and quantifying damage show that, none of the three damage indicators can provide satisfactory estimates of the presence and location of the damage, particularly for lower levels of damage. Their relatively low-sensitivity to low levels of damage leading to inaccurate predictions, particularly when data is polluted with environmental noise and seasonal changes, is also well-documented [22].

A potential improvement to the uncertainties impacting the efficacy of the aforementioned damage indicators is statistical evaluation of the variation of these indicators over time through long-term monitoring [25]. Within this context, this article proposes a framework for long-term vibration-based monitoring of bridges that aims to negate the impacts of random sources on the damage indicators. The impacts of these sources, which include but are not limited to measurement noise and environmental effects, are by nature random and transient. On the contrary, the variations in the damage indicators are permanent. The proposed framework aims to capture the permanent nature of the variation in the damage indicators due to damage while ignoring the transient variations due to random sources.

The article is structured as follows. First, the efficacy of the three damage indicators in detecting and locating damage for different damage levels and locations on a 84.2 m long, two-span railway bridge is evaluated. The damage indicators were computed from the mode shapes identified using stochastic subspace identification method from vibrations created by a simulated train-crossing. In order to replicate the effect of different sources of uncertainties on the measurements, the computed vibrations were polluted using spatially correlated noise. Second, the proposed long-term monitoring framework is described and its application on the case study is summarized. In the proposed framework, damage detection and location is based on evaluating the frequency of the chosen damage indicator exceeding the threshold value for an acceptable false alarm rate over a number of observations instead of evaluating the discrete values of the indicators. Later, impact of several factors including the damage location, damage indicator used in the framework and the noise level in the measurements, on the success of the proposed framework was investigated through numerical analysis. Finally, conclusions drawn from the study and recommendations for future work are summarized.

## 2. Vibration-Based Damage Indicators

This section describes briefly the vibration-based damage indicators, that were developed in early 1990s [22,23,24] and are widely used in vibration-based health monitoring.

### 2.1. Modal Curvature

For structures that exhibit bending behavior and conform to the assumptions of Euler-Bernoulli formulation, the bending moment at location *x*, *M(x)*, and bending stiffness, *EI*, of the beam-cross section is related through the curvature at the same location, $\nu^{\prime \prime }\left(x\right)$:

(1) $$\nu^{\prime \prime }\left(x\right)\approx \frac{M(x)}{EI}$$

where *E* is the modulus of elasticity of the material and *I* is the cross-sectional moment of inertia. Equation (1) indicates that the flexural stiffness of a cross-section is inversely proportional with the curvature. Thus, for a given moment demand, any damage that is accompanied by a reduction in the flexural stiffness of the cross-section leads to an increase in curvature [23]. Therefore, changes in curvature can be monitored to detect and locate damage. Furthermore, the extent of the damage at a section can also be estimated by measuring the amount of change in the curvature [22,27]. Higher levels of damage are expected to lead to a larger reduction in the flexural stiffness of the cross-section and, in turn, a larger increase in the curvature compared to the undamaged state.

The mode shapes detected using Experimental and Operational Modal Analysis (EMA and OMA) techniques are discretized at the sensor locations. The spatial resolution of the detected mode shapes can be interpolated using continuous functions such as cubic spline in order to improve the estimation of the location of possible damage. The curvature of mode shape *j* that is approximated at discrete points equally spaced at a distance *h* along its length can be computed using the central difference approximation to the second derivative at the degree of freedom *i*:

(2) $$\nu_{j,i}^{\prime \prime }\approx \frac{\nu_{j,i-1}-2\nu_{j,i}+\nu_{j,i+1}}{h^{2}}$$

where $\nu_{j,i}$ is the component of the jth mode shape at location *i*. The difference between the modal curvature of the possibly damaged state, *d*, and the undamaged state, *u*, is defined as the Curvature Damage Index (CDI) and can be used to detect, locate and eventually quantify any potential damage using the identified mode shapes:

(3) $$CDI_{c}=\sum_{j=1}^{nModes}\left|{\nu_{j,i}^{{}^{\prime \prime }}}^{d}-{\nu_{j,i}^{{}^{\prime \prime }}}^{u}\right|$$

### 2.2. Modal Strain Energy

Another damage index that is based on the modal features is the modal strain energy, which can be defined as the strain energy stored in a structure when it deforms purely in its mode shape pattern. It is based on the idea that the strain energy distribution throughout the structure changes with damage. More specifically, when the stiffness of one segment of the structure is reduced due to sustained damage, it can no longer absorb the same amount of energy as it did when undamaged [24]. This results in a deviation from the original strain energy distribution, which can then be used to detect and locate any potential damage.

If a beam is divided into *N* subregions, then the energy stored in each subregion *j* in the ith mode shape is given by Equation (4a) while the total energy stored in the entire beam can be computed using Equation (4b).

(4a) $$U_{i,j}=\frac{1}{2}\int_{a_{j}}^{a_{j+1}}EI_{j}{\left[\nu_{i}^{\prime \prime }\left(x\right)\right]}^{2}\;dx\;$$

(4b) $$U_{i}=\frac{1}{2}\int_{0}^{l}EI{\left[\nu_{i}^{\prime \prime }\left(x\right)\right]}^{2}\;dx\;$$

where $a_{j}$ and $a_{j+1}$ are the start and end coordinates of the subregion *j* and *l* is the length of the entire beam.

Assuming that the subregions are small enough and flexural rigidity is constant in the subregions, the fractional energy, $f_{i,j}$, defined as the ratio of the energy stored in each subregion *j* to the total strain energy stored in the entire beam can be computed as:

(5) $$f_{ij}=\frac{U_{i,j}}{U_{i}}=\frac{\int_{a_{j}}^{a_{j+1}}{\left[\nu_{i}^{\prime \prime }\left(x\right)\right]}^{2}\;dx}{\int_{0}^{l}{\left[\nu_{i}^{\prime \prime }\left(x\right)\right]}^{2}\;dx}$$

Considering all the identified modes, a damage index, $\beta_{ij}$, can then be defined for each subsegment, *j*, and mode shape, *i*, of the beam as the ratio of the fractional energy of the potentially damaged state, *d*, to the fractional energy of the undamaged state, *u*:

(6) $$\beta_{ij}=\frac{f_{ij}^{d}}{f_{ij}^{u}}=\frac{\frac{\int_{a_{j}}^{a_{j+1}}{\left[\nu_{i}^{\prime \prime }{\left(x\right)}^{d}\right]}^{2}\;dx}{\int_{0}^{l}{\left[\nu_{i}^{\prime \prime }{\left(x\right)}^{d}\right]}^{2}\;dx}}{\frac{\int_{a_{j}}^{a_{j+1}}{\left[\nu_{i}^{\prime \prime }{\left(x\right)}^{u}\right]}^{2}\;dx}{\int_{0}^{l}{\left[\nu_{i}^{\prime \prime }{\left(x\right)}^{u}\right]}^{2}\;dx}}$$

This formulation can often lead to potential problems due to very small values of the denominator. In order to eliminate these problems, the value of both the nominator and the denominator are increased by a value of 1.0. Therefore, the definition of the strain energy based damage indicator can be revised and rewritten for all identified mode shapes as [27]:

(7) $$\beta_{j}=\frac{\sum_{i=1}^{nModes}f_{ij}^{d}+1}{\sum_{i=1}^{nModes}f_{ij}^{u}+1}$$

### 2.3. Modal Flexibility

Flexibility matrix is the inverse of the stiffness matrix and is generally more straightforward to identify compared to the stiffness matrix. Damage in a structure that reduces the stiffness of the structure leads to an increase in the flexibility matrix. Damage detection using this method is based on computing the modal flexibility matrix for the undamaged and potentially damaged states of the structure from the identified mode shapes using the equation:

(8) $$\left[F\right]=\sum_{i=1}^{nModes}\frac{1}{w_{i}^{2}}{\left[\phi \right]}_{i}{\left[\phi \right]}_{i}^{T}$$

Since the contribution of each mode is multiplied by the inverse of the circular frequency of that mode, $w_{i}$, the contribution of the higher modes, which are more difficult to identify using EMA and OMA, reduces leading to a more reliable estimate of the flexibility matrix as compared to the stiffness matrix. The change in the flexibility matrix between the two states of the structure can then be computed as:

(9) $$\left[\Delta F\right]=\left[F_{d}\right]-\left[F_{u}\right]$$

For each degree of freedom *j*, the change in the flexibility matrix for that degree of freedom can be defined as the maximum absolute value of the elements in the jth column of the matrix ΔF:

(10) $$\left[\delta f_{j}\right]=max|\delta f_{ij}\left|=max\right|f_{ij}^{d}-f_{ij}^{u}|$$

The quantity $\delta f_{j}$, which is a measure of the change in flexibility between the two states of the structure can be used to detect and locate the damage.

## 3. Case Study

To evaluate the efficacy of the vibration-based parameters and the proposed framework, a case study was conducted on a 84.2-m, two-span railroad bridge located on the Ofot line in Northern Norway.

**Figure 1:**
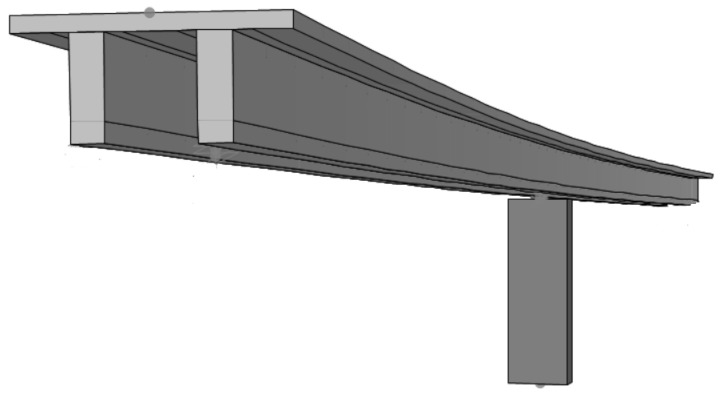
Numerical model of the bridge.

A finite element (FE) model of the bridge was developed in the CSI Bridge environment (Figure 1). Both the bridge deck and the pier were modeled as elastic beam-column elements. The bridge was assumed to be pinned at the abutments and the bottom of the pier was modeled as fixed. Although the pinned connections at the abutments may not have simulated the behavior of the bridge in the two horizontal directions, the behaviour of the bridge in the vertical direction was simulated satisfactorily as the stiffness of the elastomeric bearings are relatively high in the vertical direction. Since the scope of this study was limited to comparing the behavior of the undamaged and damaged bridge in the vertical direction, the limitations associated with modeling of the boundary conditions did not impact the results of the study. The results presented in the upcoming sections are based on the numerical analysis conducted on this finite element model.

**Figure 2:**
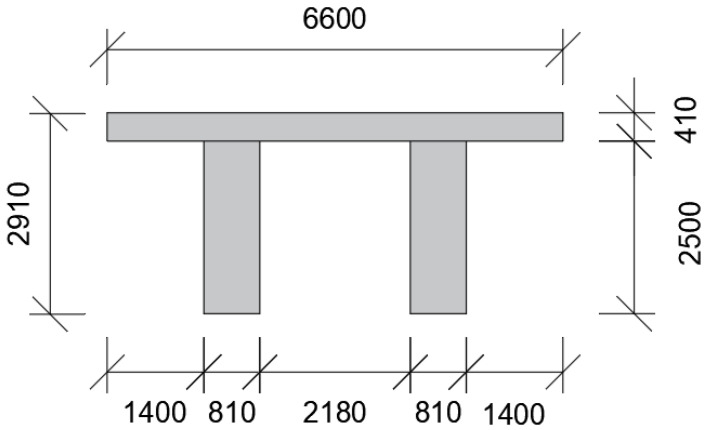
Cross-section of the bridge deck. All dimensions are in mm.

The deck of the bridge was a prestressed concrete double-tee section with a total depth of 2910 mm; see Figure 2. The deck housed a single-track railway line that was trafficked heavily by iron-ore trains that carries the iron ones mined in Kiruna, Sweden to the port of Narvik in Norway.

**Figure 3:**
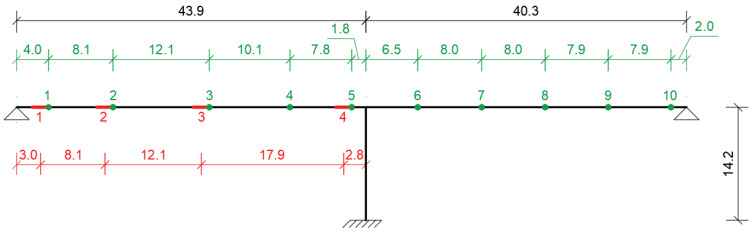
Overview of sensor locations indicated with green points and the damage locations indicated with red line segments. All dimensions are in m.

The single pier was located very close to the middle of the bridge dividing the bridge into two spans of 43.9 m and 40.3 m. The elevation view of the bridge that shows the overall bridge dimensions is shown in Figure 3. The pier was a rectangular reinforced concrete section with dimensions of 1300 mm × 3600 mm. At the top, the pier was rigidly connected to the deck. At each abutment, the deck sat on two elastomeric bearings. In order to emulate a realistic SHM system, a total of 10 sensor locations, five at each span, were selected. For each span, the first two of the five sensors were placed close to the abutment and the pier, respectively. The sensor locations are depicted by green points on the bridge in Figure 3 together with the four simulated damage locations, which are represented by the red line segments.

## 4. Damage Scenarios, Simulated Vibrations and System Identification

A total of 20 damage scenarios distributed over four damage locations, which are marked in Figure 3 with red lines, and five separate damage levels were simulated in the study. The damage locations were selected carefully at different parts of the bridge in order to be able to evaluate the efficacy of the damage indicators and the proposed framework in detecting the damage at different locations including the middle of the span as well as close to the abutment and the pier.

One of the premises of SHM applications is its potential to be able to detect damage at its initial stages. In order to be able to systematically evaluate the sensitivity of the damage indicators to different levels of damage as well as the efficacy of the developed framework in detecting various levels of damage, a total of five damage levels were introduced at each damage location. These damage levels were simulated by decreasing the flexural stiffness of a 2-m long segment of the bridge deck by 5%, 10%, 20%, 50%, and 80% at the desired location. These five damage levels also simulate the development of damage over time once initiated and will be referred to as 5%, 10%, 20%, 50%, and 80% damage levels, respectively in the following sections.

It should be noted that the simulated damage levels may emulate different physical damage states for different damage locations and bridge types. While a reinforced concrete bridge was used as a case study, the proposed framework is equally applicable for steel or composite bridges. For example, 5% damage for concrete bridges may represent micro-cracks in concrete for reinforced concrete bridges while the same damage level may represent cracking in the weld connections for steel bridges. In other words, 5% damage level did not represent one specific physical damage case but emulates all possible cases which resulted in a 5% decrease in the flexural stiffness of the bridge deck. Similarly, 10%, 20%, 50%, and 80% damage levels represented any physical damage level that led to a 10%, 20%, 50%, and 80% decrease in the flexural stiffness (i.e., EI value) of the bridge deck, respectively.

**Figure 4:**
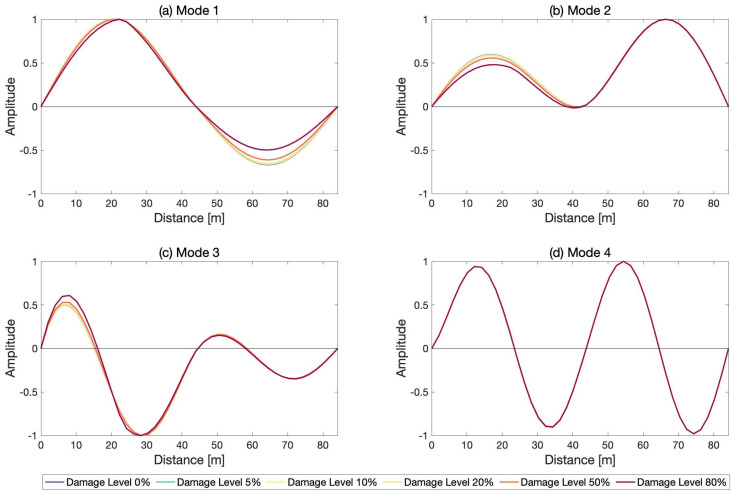
Mode shapes of the first four modes of the undamaged bridge and the bridge damaged at the middle of the first span.

Eigen-value analysis was conducted to compute the modal parameters of the bridge both in its undamaged and damaged states. Figure 4 depicts the first four mode shapes of the bridge in the vertical direction along with the mode shapes computed for the bridge assumed to be damaged at the middle of the first span (damage location 3 in Figure 3) for different damage levels.

Instead of working directly with the mode shapes computed using the FE model and introducing spatially non-correlated random noise on the mode shapes, a more realistic approach was used to emulate the entire SHM process. For this, vibrations were generated on the FE model through moving load analysis that simulates train crossings on the bridge. HSLM-A10 train was modeled in the FE model as moving load and was virtually driven on the bridge with a speed of 80 km/h for both the undamaged state of the bridge as well as all 20 damage scenarios. The accelerations recorded at the 10 virtual accelerometers (Figure 3) were then used to identify the modal properties of the bridge at both its undamaged and damaged states using operational modal analysis techniques.

In addition to the ideal case where no noise in the measurements were considered, four separate noise levels were introduced to the simulated vibrations: 1%, 5%, 10%, and 20% noise. Measurement noise was simulated by polluting the accelerograms recorded at each virtual sensor using:

(11) $${\ddot{u}}_{j,k}={\ddot{u}}_{j}^{0}+\beta \lambda {\ddot{u}}_{j}^{0}$$

where ${\ddot{u}}_{j}^{0}$ is the non-noisy acceleration record at sensor *j*, ${\ddot{u}}_{j,k}$ is the kth noisy sample of the acceleration record at the same sensor, β is a parameter that determines the level of noise and λ is a random number with a standard normal distribution. The parameter β emulates uncertainties that affect acceleration records in real-life applications including but not limited to measurement errors, sensor sensitivity, quality of the hardware used, and environmental factors. In order to simulate the four noise levels, β parameter was set to 1%, 5%, 10%, and 20%, respectively.

This approach has two distinct benefits compared to using the mode shapes obtained directly from modal analysis. First of all, this approach takes into account the uncertainties related to the identification of the mode shapes from the recorded accelerograms using OMA whereas using the computed mode shapes directly ignores these uncertainties. Secondly, as discussed in [28], polluting the simulated response using spatially correlated noise as used in this study provides a much improved emulation of the reality compared to spatially non-correlated noise, which leads to non-continuous mode shapes.

For the undamaged state, a total of 1000 set of accelerograms were created for each noise case by polluting the non-noisy accelerograms obtained from the FE analysis using Equation (11). The first 500 sets were used to identify the threshold values that will ensure that the desired rate of false alarm is not exceeded for each sensor location.

Due to the sensitivity of the mode shapes and the vibration-based damage indicators to random sources, a threshold value can be defined for a pre-determined false alarm rate so that if the variation of the damage indicator exceeded the threshold, the variation was judged to be due to damage. Otherwise, the variation in the damage indicator was assumed to be due to random sources. Ref. [25] provides a detailed description and statistical background of the threshold value computation.

The remaining 500 sets of accelerograms from the undamaged state emulated the phase of the monitoring where the bridge remained undamaged.

**Figure 5:**
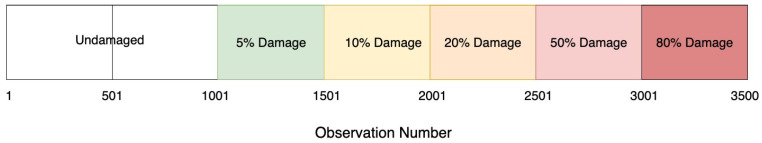
Development of damage with observation number. Each observation represents a set of polluted accelerograms.

The development of damage over time was simulated through generating 500 sets of polluted accelerograms for each damage level, location and noise level using the noise-free accelerograms obtained from the FE analysis and Equation (11). For each damage location and noise level, each polluted set of accelerograms emulated an observation conducted in a real SHM study and was given an observation number. The damage level was assumed to increase at every 500th observation starting from observation number 1001 emulating the development of the damage over time. Figure 5 presents the development of the simulated damage at every damage location with respect to the observation number.

Stochastic subspace identification with covariance (SSI-cov) [29] method was used to identify the modal properties of the non-noisy and noisy accelerograms. Once the modal values at the 10 virtual sensor locations were identified using the SSI-cov method, the spatial resolution of the identified mode shapes was increased by fitting a cubic spline function.

**Figure 6:**
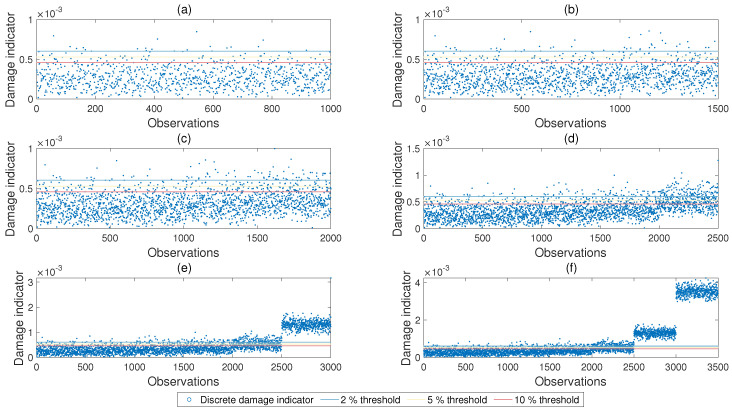
Scatter plot of the modal curvature damage indicator at sensor 3 for damage at the mid-span; 5% noise. Subplots (**a**–**f**) show the development of the scatter plot from the first 1000 observations to 3500 observations. The damage level increases with the observation number as summarized in Figure 5.

The damage indicators summarized in Section 2 were then computed using the identified mode shapes for each observation. As an example, Figure 6 depicts the scatter plot of the modal curvature damage indicator at sensor 3 for damage at the middle of the first span for each of the 3500 observations.

## 5. Evaluation of Efficacy of Damage Indicators for Discrete Observations

In order to evaluate the efficacy of damage indicators summarized in Section 2 in detecting damage for discrete observations, the probability of successfully detecting damage for each damage scenario was computed as the ratio of the number of cases where the damage indicator was greater than the predetermined threshold value to the total number of cases for each damage scenario.

**Figure 7:**
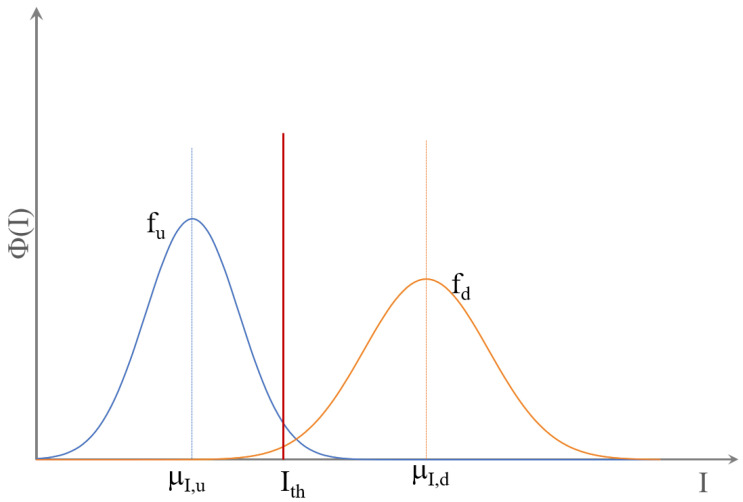
Distribution of the damage indicator in the undamaged and damaged states.

For this, first the threshold values for each damage indicator for different acceptable false alarm rates were computed. Due to noise and other uncertainties in the measurement process, the damage indicators provided non-zero values for the undamaged bridge. Figure 7 presents the distribution of the damage indicator, I, for the undamaged and damaged states. The function $f_{u}$ describes the distribution of the damage indicator for the undamaged state for a population of observations, while the distribution $f_{d}$ shows the distribution of the indicator for the damaged state. The variation in the damage indicator for the undamaged state was a result of random variations in the indicator such as measurement noise and environmental factors. Damage, on the other hand, led to a permanent increase in the damage indicator and resulted in a shift in the distribution. The threshold value was defined so that if the variation of the damage indicator exceeded the threshold, the location was assumed to be damaged [25]. The threshold value of the damage indicator, $I_{th}$ for a given acceptable false alarm rate of γ was computed using Equation (12). The probability of successfully detecting damage, $P_{d}$, could be defined as the area under the distribution for the damaged state, $f_{d}$, starting from the threshold value $I_{th}$; Equation (13).

(12) $$\int_{I_{th}}^{\infty }f_{u}dI=\gamma$$

(13) $$P_{D}=\int_{I_{th}}^{\infty }f_{d}dI$$

In this case study, in order to evaluate the range of the damage indicators for the undamaged bridge, for each damage location and noise level, the damage indicators computed for the first 500 observations were plotted; see Figure 6a. The threshold values of the damage indicator for an acceptable false alarm rate was computed such that the ratio of the number of cases that were above the threshold value to the total number of observations (500 in this case) was equal to the desired false alarm rate; Equation (12). Threshold values for the modal curvature damage indicator for sensor number three for 2%, 5%, 10% acceptable false alarm ratios computed using the methodology described above are also shown in Figure 6.

**Figure 8:**
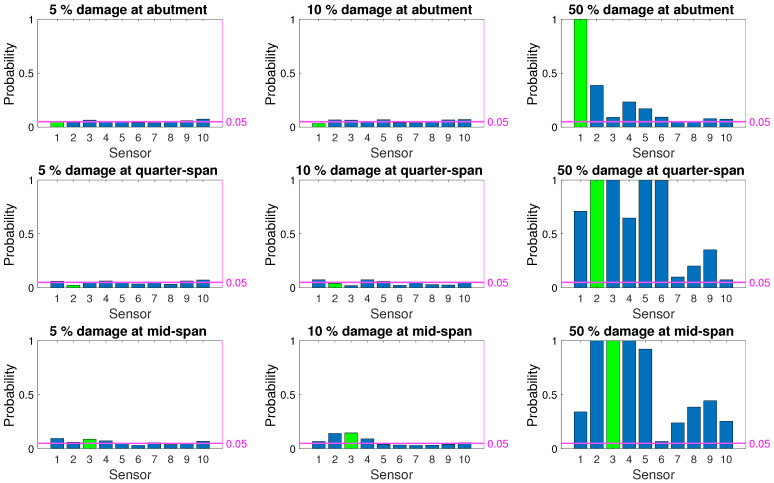
Probability of damage detection using modal curvature damage indicator for 5% noise.

**Figure 9:**
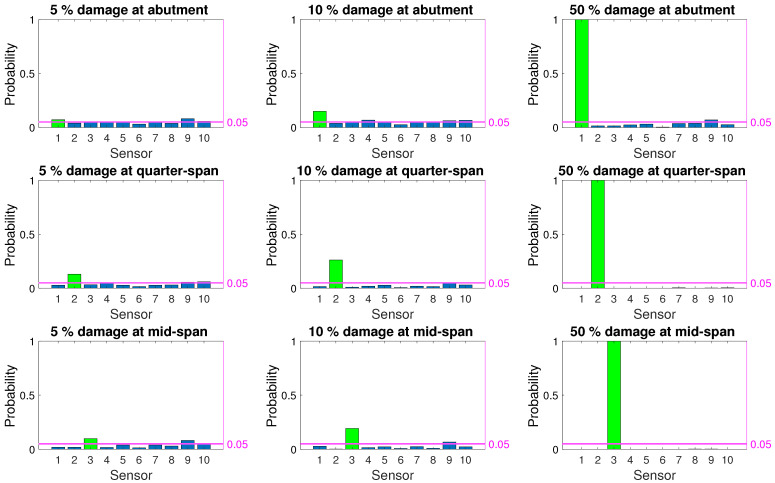
Probability of damage detection using modal strain energy damage indicator for 5% noise.

**Figure 10:**
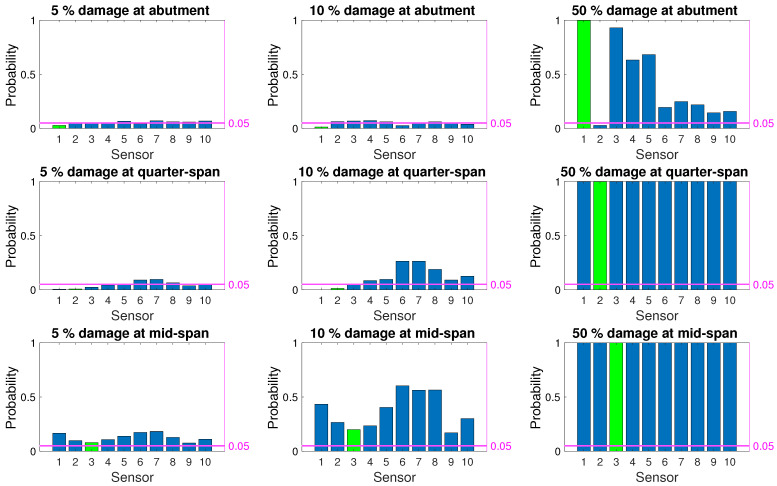
Probability of damage detection using modal flexibility damage indicator for 5% noise.

Figure 8, Figure 9 and Figure 10 depict the probability of detection at each sensor for 5%, 10%, and 50% damage levels at the abutment, quarter-span and mid-span (damage locations 1, 2, and 3 in Figure 3) for the three damage indicators; modal curvature, modal strain energy and modal flexibility, respectively. The noise level was kept constant at 5% for all three figures. It should be noted that the probability of detection at a sensor location where there was no damage indicates the probability of false alarm for that location. In Figure 8, Figure 9 and Figure 10, the correct damage location is indicated by the green column, whereas the blue columns indicate the probability of detection at the locations where there was no damage.

Figure 8 shows that the modal curvature damage index had a very low probability of correctly detecting damage at all three locations for low damage levels. Furthermore, for 5% and 10% damage levels, modal strain energy also failed to provide a correct detection rate that is above the threshold for false alarm; see Figure 9. Modal flexibility damage indicator provided a better chance of detecting damage for 10% damage level, especially when the damage was located at the quarter-span and mid-span. However, the accuracy of locating the detected damage for low damage levels using the modal flexibility parameter remained very low as the damage was, more often than not, detected at an incorrect location; see Figure 10. When the damage level increased to 50%, all damage indicators could consistently detect damage. However, modal strain energy (Figure 9) separated itself from the other damage indicators in locating damage as it provided a 100% success rate at detecting the damage at the correct location while no damage was detected on the other locations. Modal curvature and modal flexibility damage indicators, on the other hand, detected damage in several other locations in addition to the correct location for 50% damage level.

**Figure 11:**
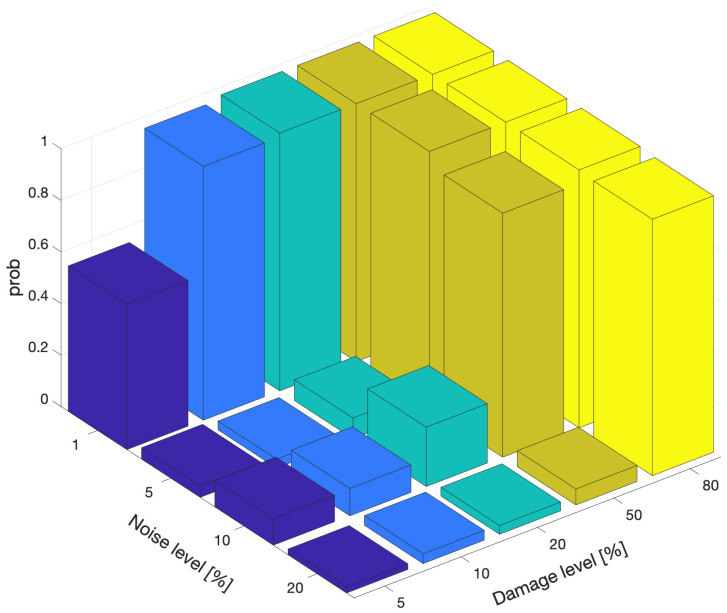
Effect of noise on probability of detecting damage at the abutment using modal curvature damage indicator.

A parameter that has the potential to impact the probability of successful damage detection and localization is noise. In order to evaluate the impact of noise, the probability of correctly detecting damage at the abutment using the modal curvature damage indicator using three modes for different damage and noise levels is depicted in Figure 11. The figure clearly shows that, noise had a significant impact in successfully detecting damage. For 1% noise level, the damage indicator could very successfully detect damage starting from a damage level of 10%. On the contrary, when the noise level increased, the probability of detecting damage fell drastically even for higher damage levels such as 50%. It should be noted here that 1% noise could be adjudged to be a very low level of noise while 20% could be assumed to be very high. For more realistic noise levels of 5% and 10%, the success of detection remained very low for lower damage levels, i.e., 5% to 20% damage. Although, the effect of noise is presented for only one damage indicator and location for brevity, the results obtained for the other damage indicators and locations were very similar to Figure 11 and the observations drawn from this figure could be generalized.

The results presented in this article, along with others [22,25] clearly depicted the shortcomings of the vibration-based damage indicators in detecting and locating damage, especially for lower damage levels, when the damage indicator was based on two discrete observations. Despite their physical connection to damage, these damage indicators suffered from low sensitivity to damage, high sensitivity to noise, and the smoothing effect of the polynomial fitted to increase the spatial resolution of the discrete mode shape data [22] leading to low success rates in detecting and locating damage.

## 6. Proposed Framework

As summarized in Section 5 as well as in literature [8,12,16,22,25], none of the damage indicators provide satisfactory results in terms of detecting low levels of damage when the evaluation is based on direct comparison of observations at two discrete points in time; the first point in time being the known undamaged state while the other is the potentially damaged state. Particularly, the low level rate of successful detection at the initial stages of damage formation, i.e., low damage levels as indicated by Figure 8, Figure 9 and Figure 10, render these damage indicators unsuitable for SHM applications which aim to detect damage at earlier stages when the evaluation is based on two discrete observations in time. On the other hand, all three damage indicators maintain their attractiveness in SHM applications due to their physical theoretical connection to damage.

In this study, we propose a long-term monitoring framework that aims to eliminate the shortcomings of vibration-based damage indicators in identifying and locating low levels of damage while retaining their physical connection to damage. For this, we propose a new parameter that can be used together with any damage indicator and is based on a population of observations as opposed to discrete observations.

(14) $$freq=\frac{n(DI>TH)}{n_{pop}}$$

In Equation (14), DI is the damage indicator that will be used in the framework, TH is the threshold value associated with the acceptable false alarm rate, and $n_{pop}$ is the number of observations that will be used in damage detection. The computed damage parameter, freq, indicates the number of cases, out of the last $n_{pop}$ observations, where the damage indicator exceeds the threshold value. In other words, it is the frequency of the damage indicator exceeding the threshold value computed from the last $n_{pop}$ observations.

In Figure 6d, the values of the modal curvature damage indicator for each observation is plotted for damage levels of 5%, 10%, and 20% for observations 1001 to 1500, 1501 to 2000, and 2001 to 2500, respectively. This figure clearly shows that the probability of correct detection remains low when each point on the figure is separately evaluated by comparing each value to the threshold value. On the other hand, it can also be observed that, as the damage level increases, the number of cases exceeding the threshold value increases. The proposed freq parameter aims to benefit from this increase and utilizes a population of the observations shown in Figure 6 rather then the individual observations independently from each other. Continuing to use the Figure 6d as an example and assuming that the number of observations used in computing the freq parameter is $n_{pop}$, the freq parameter at each observation *n* can be computed as the ratio of the number of points in the last $n_{pop}$ observations prior to and including observation *n* exceeding the threshold value to the value of $n_{pop}$.

In Equation (14), the population size is a variable and can be chosen by the user for each case depending on the frequency of data collection and availability of data. In this study, the population size was selected as 100; i.e., the frequency of exceedance of the threshold value was computed from the last 100 observations for all cases.

The proposed framework, which is anchored at the freq parameter given in Equation (14), is composed of the following steps.

1. Training period: During this period, the threshold values for acceptable false alarm rates will be determined. For this, the structure will be monitored for a certain period from the start of the monitoring program and the values of the selected damage indicator will be computed during this period. At the end of this period, the structure will be inspected to confirm that no changes in the condition of the structure occurred during the training period. The variation in the damage indicators during this period will provide information regarding the impact of the random factors other than damage on the damage indicators. This data will then be used to compute the threshold values for the acceptable false alarm rates using the method described in Figure 7 and formulation given in Equation (12). In this study, the first 500 observations (Figure 5) were used as the training period.
2. Monitoring period: After the completion of the training period, modal parameters of the potentially damaged structure will be computed from the measured accelerations periodically. Each of these periodic computations will constitute an observation and will be used to compute the freq parameter. The damage indicator that will be used in the framework (i.e., modal curvature, modal strain ratio or modal flexibility) will be computed from the identified mode shapes and recorded in a database. The frequency of the damage indicator exceeding the threshold value, i.e., the freq parameter, will then be computed using the last $n_{pop}$ observations (Equation (14)) and plotted against the observation number. It should be noted that the freq parameter and the threshold value, although not directly comparable, are closely related to each other as both are computed from the distribution of the damage indicator in the undamaged and potentially damaged states. For the undamaged state, the freq parameter is expected to remain virtually constant around the threshold value while systematically increasing with an increase in the damage level.

Therefore, in the proposed framework, the damage is identified not through a single observation of the damage indicator exceeding the threshold value but through a gradual increase in the number of observations where the indicator starts to systematically exceed the threshold value. Considering that variations in damage parameters such as modal curvature and modal strain energy due to reasons other than damage is random, using a population of observations is expected to negate the impacts of such random variations. Contrary to variations due to random sources, variations in the vibration-based damage indicators due to damage are permanent and expected to be visible when a population of observations is considered to detect and locate damage.

### 6.1. Application

The developed framework was applied to the case study using the simulated vibrations summarized in Section 4. The first 500 observations for each noise level were used as the training period to determine the threshold values for each of the three damage indicators for three different acceptable false alarm rates: 2%, 5% and 10%. The monitoring period started from observation number 501 and the following damage levels were induced to the structure over time: between observations 501 and 1000, the structure remains undamaged. This part was used to verify the robustness of the proposed framework against false alarms. The 5% damage was introduced starting from observation 1001 and increased gradually as described in Section 4 and shown in Figure 5. The gradual increase in the simulated damage emulated the development of damage on a structure from its early stages (i.e., 5% reduction in flexural stiffness) to very high levels (i.e., 80% reduction in flexural stiffness) and was used to evaluate the success of the proposed framework to detect damage at every stage of the damage development.

**Figure 12:**
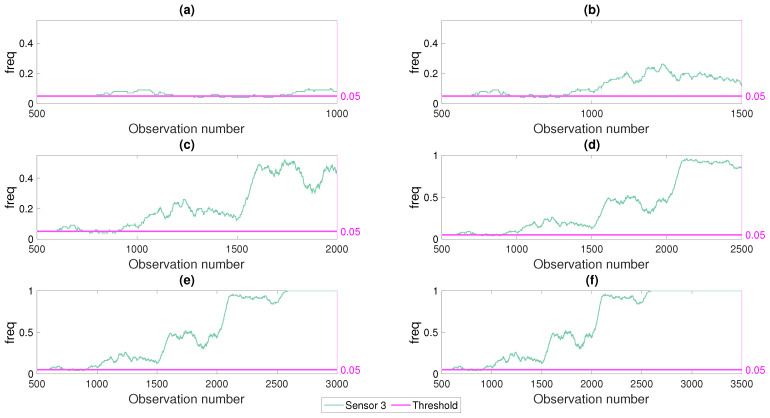
Development of the frequency parameter with damage and observation number for sensor number 3. Subplots (**a**–**f**) show the development of the scatter plot from the first 1000 observations to 3500 observations. The line at 0.05 for all cases indicates the frequency threshold for acceptable false alarm rate.

Figure 12 presents the progress of the monitored freq parameter with time (i.e., observation number) for different damage levels for a noise level of 5%. Modal curvature at sensor number 3 was used as the damage indicator and damage was introduced at the middle of the first span (damage location 3 in Figure 3). In Figure 12a, the structure was undamaged between observations 501 and 1000 and the freq parameter computed using the modal curvature (Equation (2)) at the sensor closest to the damage location (sensor #3) remained under or very close to the threshold value. As shown in Figure 12b, the freq parameter crept over the threshold value as 5% damage was introduced at the damage location 3. It should be noted that during the first 100 observations after the damage was introduced (between observations 1001 and 1100), the freq parameter increased gradually because the computation of the freq parameter included values from both undamaged and damaged cases as the last 100 observations were used in the computation. After observation number 1100, the freq parameter was computed solely from the observations at the damaged state and, therefore, became stable until the damage level increased to 10% starting from observation number 1501; Figure 12c. Figure 12c,d follow a similar pattern where the freq parameter started to increase immediately after the damage level increased followed by flattening at a constant level. On the other hand, when the damage level reached 50%, the frequency of the modal curvature damage parameter exceeding the threshold value saturated at 100%; Figure 12e. Hence, the freq parameter did not change when the damage level increased from 50% to 80%.

Figure 12 depicts that the monitored freq parameter, which is the frequency of the selected damage indicator exceeding the threshold for a population of observations, was very effective in following the development of damage. Using a population of observations over time had the potential not only to eliminate the false alarms by negating the impact of random variations in the modal curvature damage parameter, but also to help quantify the damage level as the variation of the freq parameter provided a clear indication of the level of damage, especially at the lower damage levels. More specifically, the gradual increase in the freq parameter with the introduction of low levels of damage (5% and 10% reduction in flexural stiffness; Figure 12b,c) followed by a period where the freq parameter stayed flat clearly indicated the points where the level of damage increased; i.e., observation number 1001 and 1501. Recalling that reliable detection of lower levels of damage proved to be the most challenging task as long as use of the damage indicators based on two discrete points in time was concerned (Figure 8, Figure 9 and Figure 10), the proposed monitoring framework that was based on the freq parameter provided a very attractive alternative for detection and quantification of low levels of damage.

In the following paragraphs, first, the efficacy of different damage indicators in predicting damage when used within the proposed framework is investigated. Afterwards, impact of different parameters including the noise and number of mode shapes used in the computation of the damage indicator on the results of the developed framework is investigated. Finally, the success of the developed framework in predicting damage at different locations on the bridge is explored.

#### 6.1.1. Effect of Damage Indicator

**Figure 13:**
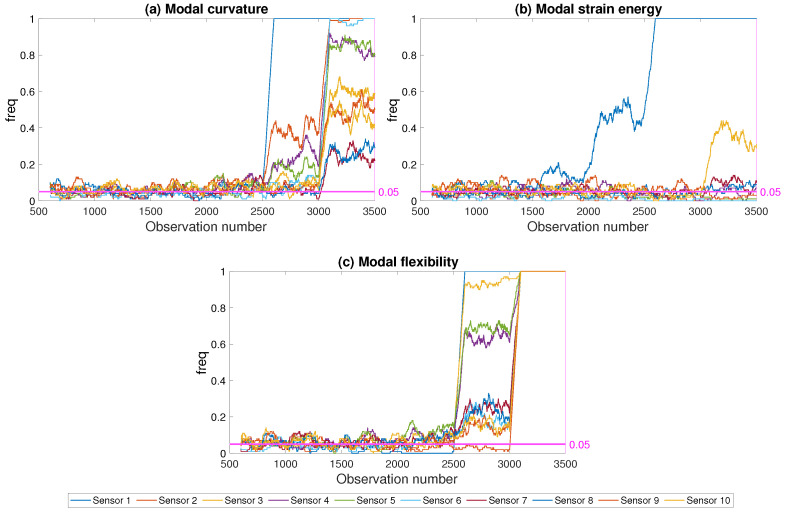
Effect of damage indicator; damage at the abutment; 5% noise.

**Figure 14:**
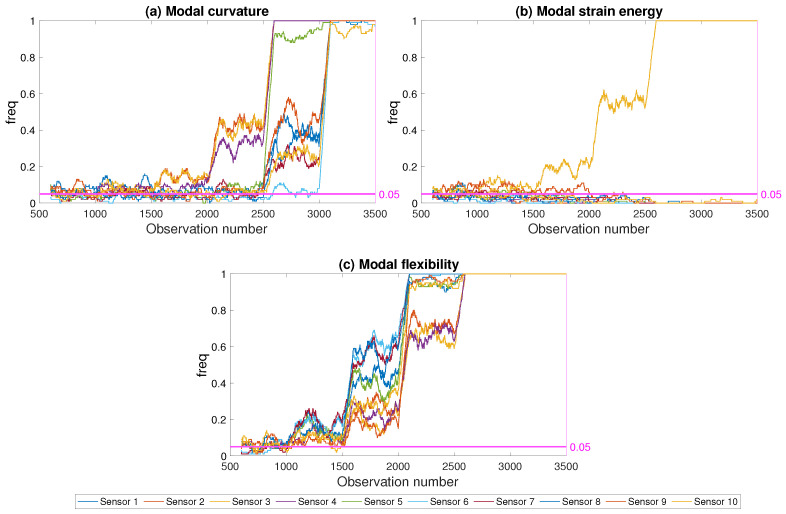
Effect of damage indicator; damage at the mid-span; 5% noise.

Three different damage indicators were used in computing the freq parameter in the framework to determine the impact of the indicator on the success of the framework: modal strain energy, modal curvature and modal flexibility. Figure 13 and Figure 14 depict the results for damage scenarios at the mid-span and the abutment for the three damage indicators, respectively, for a noise level of 5%. The freq parameter was computed at each sensor in order to evaluate the success of the developed framework in locating damage. It should be noted that, in Figure 13 and Figure 14, along with all the similar figures that will be presented in the coming sections, all damage levels were represented as the damage level increased with the observation number at every 500th observation starting from observation number 1000; see Section 4 and Figure 5 for details.

For the damage in the abutment (damage location 1 in Figure 3), the closest sensor to the damage location was sensor number 1, while for the damage at the middle of the first span (damage location 3 in Figure 3), the closest sensor to the damage location was sensor number 3. For the modal curvature and the modal flexibility damage indicators, the developed framework could successfully predict the damage at the middle of the first span starting from relatively low levels, Figure 14. However, both damage indicators failed to provide a very clear picture of the damage location as the freq parameter computed using modal curvature and modal flexibility parameters exceeded the threshold at several sensors concurrently. On the other hand, both damage indicators struggled to detect low levels of damage when the damage was close to the abutment, i.e., between observations 1000 and 2500 in Figure 13, where the damage at abutment varied between 5% and 25%.

Contrary to modal curvature and modal flexibility, modal strain energy provided very clear indication of the damage location for both cases; i.e., damage at the abutment and damage at the mid-span, when used within the developed framework. The freq parameter increased with increasing damage at only the sensor closest to the damage location while the freq values at other sensors remained below the threshold even for highest level of damage when the modal strain energy is used. Furthermore, the frequency of modal strain energy exceeding the threshold increased significantly already at 10% damage level, i.e., after observation number 1500, for both damage locations; see Figure 13b and Figure 14b.

Figure 13 and Figure 14 clearly demonstrate that the developed framework, although it could be used with any damage indicator, provided superior results when used together with the modal strain energy in terms of damage detection and localization. In particular, modal curvature and modal flexibility damage indicators struggled with locating the damage while modal strain energy provide a clear picture about the damage location starting from relatively low levels of damage. Although the results are presented for two damage locations and one noise level for brevity, the results were very similar for all damage locations and noise levels considered in the study. As such, the evaluation of the developed framework in the following paragraphs will be based on the modal strain energy damage indicator.

#### 6.1.2. Effect of Number of Modes

**Figure 15:**
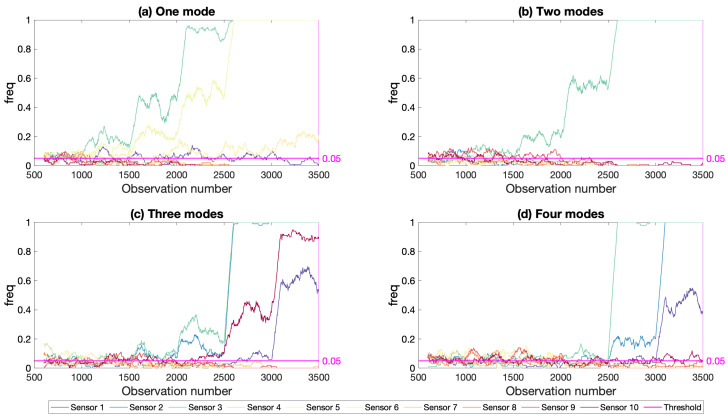
Effect of number of modes; damage at the mid-span; 5% noise.

As summarized in Section 2, all three vibration based damage indicators could be computed using a different number of modes depending on the structure and the user’s preference. The number of mode shapes used in computing the damage indicators was previously shown to significantly impact the success of the indicators in detecting and locating damage [22]. In order to evaluate the impact of the total number of mode shapes included in the damage indicator used in the the proposed long-term framework, the framework was applied to the scenario where the damage was induced at the middle of the first span (damage location 3 in Figure 3) using one, two, three, and four mode shapes, respectively. The results plotted for 5% noise and using the modal strain energy damage indicator in Figure 15 indicate that increasing the number of modes included in the analysis did not necessarily lead to an improvement in damage detection and location. On the contrary, the best results were obtained for the case where the damage indicator was based on one mode or two modes; see Figure 15a,b. For both cases, the freq parameter started to increase already starting from 5% to 10% damage level (observation number 1001). On the other hand, when the damage indicator was computed using three or four modes, the developed framework could start to detect the damage successfully starting from 20% and 50% damage levels, respectively; see Figure 15c,d. The reason for the drop in the success of the developed framework can be attributed to the fact that the modal value for the third and fourth mode shapes were relatively low or very close to zero at the middle of the first span (Figure 4) leading to an insensitivity of these mode shapes to damage at this location. As such, the third and fourth modes were not as significantly impacted by the damage at the middle of the first span compared to the first two modes. Therefore, including these mode shapes negatively impacted the success of the developed framework in detecting damage, especially for low levels of damage. This observation was in line with the observations in [22] which, as a result of their work on the I-40 Bridge, concluded that, when damage is not severe, inaccurate predictions may be made if modes not significantly impacted by the damage are included in computation of the damage indicators. Considering that the higher modes has more nodes and more points that had very small modal values compared to the lower modes, they were more susceptible to insensitivity to damage at several locations compared to lower modes. Therefore, it can be concluded that using one or two modes in computing the damage indicators leads to to better damage detection and localization for low levels of damage. Hence, the rest of the article will be based on the results obtained using two mode shapes.

#### 6.1.3. Effect of Damage Location

**Figure 16:**
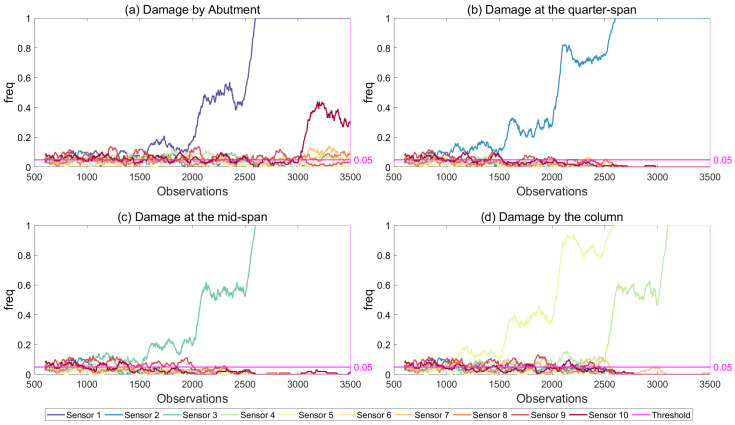
Frequency of exceedance using modal strain energy with two modes for all damage locations, 5% noise and 5% threshold.

In order to evaluate the efficacy of the developed framework in detecting and locating the damage at various locations on the bridge, four different damage scenarios were investigated. In these four scenarios, damage was introduced (i) at the abutment, (ii) at one-fourth of the length of the first span, (iii) at the middle of the first span and (iv) by the bridge pier; damage locations 1 to 4 in Figure 3, respectively. The sensor locations closest to the damage locations are shown in Figure 3 as well. As in the previous sections, the damage level was increased from 5% to 80% at every 500th observation starting from observation number 1001. The frequency of exceedance of threshold value of 5% using modal strain energy for 5% noise level is plotted in Figure 16. The results clearly indicate that, the developed framework, when used together with the modal strain energy damage indicator, was very successful in detecting and locating the damage for all four damage locations investigated. Even for 10% damage, the freq parameter clearly exceeded the threshold value at the sensor that was closest to the damage location for all damage locations while it remained below or around the threshold for all the other sensor locations.

#### 6.1.4. Effect of Noise Level

**Figure 17:**
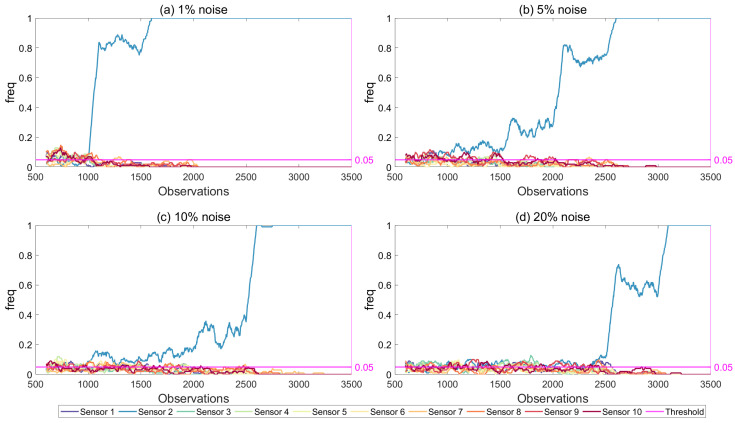
Frequency of exceedance using modal strain energy with two modes for different noise levels, increasing damage at quarter-span (location 2) and 5% threshold.

**Figure 18:**
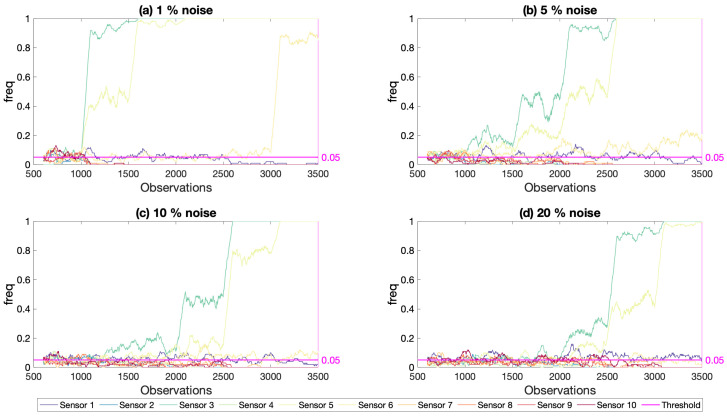
Frequency of exceedance using modal strain energy with two modes for different noise levels, increasing damage at mid-span (location 3) and 5% threshold.

Four different noise levels were included in the analysis to study the effect of noise in the results of the proposed framework: 1%, 5%, 10%, and 20%. Figure 17 and Figure 18 present the results for different noise levels for damage simulated at the one-fourth of the first span and at the mid-span; damage locations 2 and 3 in Figure 3, respectively. Both figures, which were created using the modal strain energy damage indicator, show that the proposed framework could successfully detect damage even for low damage levels at noise levels up to 10%. For the 20% noise level (Figure 17d and Figure 18d), which can be deemed to be a very high level of noise, the developed framework could only detect damage once the damage level reached 50% (observation number 2500 and beyond) when the damage was at one-fourth of the first span and 20% damage (observation number 2500 and beyond) when the damage was at the mid-span. On the other hand, it should be noted that, even for such high levels of noise, the developed framework did not produce any false alarms as the freq parameter never increased above the threshold value at the locations where there was no damage. Furthermore, for all noise levels, the developed framework could successfully locate the damage as the freq parameter increases beyond the threshold only at the sensor location closest to the damage for the damage at the quarter-span and the two closest sensor locations to the damage for mid-span damage.

It should however be noted that, for very low noise levels, the freq parameter saturated at 1.0 even at very low levels of damage rendering the quantification of damage very challenging based on the freq parameter. However, for higher, and arguably more realistic levels of noise for practical applications, the development of the freq parameter with the number of observations provided opportunities for quantification of the damage.

## 7. Conclusions

This article proposes a framework for long-term monitoring of bridges based on vibration-based damage indicators. First, the efficacy of three damage indicators that are physically connected to damage; modal curvature, modal strain energy, and modal flexibility in detecting and locating damage when used by comparing the indicator computed for each observation with a threshold value is evaluated. Later, a new framework and a parameter for detecting and locating damage that is based on monitoring the selected damage indicator for a population of observations is proposed. The efficacy of the proposed framework is verified by applying the framework on a numerical model with simulated damage at different locations of a railway bridge for varying noise levels. The effect of several parameters on the results of the proposed framework is evaluated. As a result of the numerical analysis conducted, the following conclusions were drawn:

- Modal curvature, modal strain energy and modal flexibility damage indicators have very low success rates in detecting and locating damage, especially for low levels of damage, when the estimations are based on comparing the value of the indicator from a single observation with a threshold value.
- The proposed long-term monitoring framework, that relies on monitoring the frequency of exceedance of the threshold value within a population of observations, provide an opportunity to successfully detect, locate and track the formation of damage starting from very low levels of damage.
- The proposed framework is very efficient in negating the impact of random sources that create variations in the damage indicators that are transient. On the other hand, the impact of damage on the damage indicators that is permanent are retained and highlighted through the proposed framework leading to successful detection and localization of damage at all noise levels starting from very low damage levels.
- Although the proposed framework can be used with any damage indicator, the analysis results clearly indicate that modal strain energy, by far, provides the best alternative among the three investigated indicators in terms of detecting and locating damage.
- Increasing the number of modes included in the computation of the damage indicator does not necessarily increase the success rate. On the contrary, including the third and fourth modes in computing the damage indicator, which have more number of points that has modal values close to zero, leads to a poorer performance compared to their counterparts computed using only the first one or two mode shapes.
- The proposed framework provides a very clear picture of damage development even for very high levels of noise.

This articles provides a framework that negates the effects of random variations on vibration-based damage indicators that hinder the success of these indicators, which otherwise are instinctive and based on theoretically sound formulation. The proposed framework proves also to be a promising method for damage quantification. Further studies are required in order to understand and quantify the correlation between the frequency of exceedance of the threshold for a given damage indicator and the damage level and location.

Although using long-term, continuous data instead of a limited number of observations leads to a much improved damage detection and localization, it is economically very challenging to provide a long-term monitoring setup for every bridge. It should be expected that, for the foreseeable future, monitoring of numerous bridges will be based on periodic deployment of instruments on the bridge and, hence, only the damage detection methods that are based on two discrete observations in time will be available for these bridges. Furthermore, the efficacy of the proposed framework depends on the availability of a population of observations where the damage propagation is gradual and continuous. For certain cases, where the damage initiation and propagation are very rapid, the proposed framework, which relies on a population of observations at the damaged state, may not provide reliable results due to insufficient number of observations at a given damage state. For such cases, methods that rely on discrete observations may provide more useful information due to their reliance on only two observations in time. Therefore, further research that will improve the performance of the damage indicators when based on discrete observations is needed.

Finally, the efficacy of the proposed framework should be evaluated using experimental measurement data, either using laboratory experiments of long-term monitoring data, where a population of vibration data is available for various damage levels.

## Data Availability

Not Applicable.
